# The efficacy of high-power short-duration radiofrequency for atrial fibrillation ablation: A protocol for systematic review and meta-analysis

**DOI:** 10.1097/MD.0000000000025569

**Published:** 2021-04-23

**Authors:** Peng Zhang, Ling Ma, Fei Wang, Liang Shi

**Affiliations:** Department of Cardiology, People's Liberation Army 940th hospital, Lanzhou, China.

**Keywords:** atrial fibrillation, high-power short-duration radiofrequency, meta-analysis, protocol, pulmonary vein isolation

## Abstract

**Background:**

Studies comparing data between the high-power short-duration radiofrequency (HPSR) and low power longer duration therapy were limited and inconsistent. Therefore, we conduct a high-quality systematic review and meta-analysis to assess the efficacy and safety of HPSR on outcomes for patients with atrial fibrillation (AF).

**Methods:**

The online literature is searched using the following combination of medical subject heading terms: “high-power” OR “high power” AND “radiofrequency” AND “atrial fibrillation.” MEDLINE (PubMed), EMBASE (OVID), Cochrane Central Register of Controlled Trials (CENTRAL), and Web of Science (ISI database) will be searched without any language restrictions. All clinical trials to assess the efficacy and safety of HPSR in the treatment of atrial fibrillation will be considered eligible for analysis. The present study will be performed by Review Manager Software (RevMan Version 5.3, The Cochrane Collaboration, Copenhagen, Denmark). Ethical approval and patient consent are not required because this study is a literature-based study.

**Results:**

This study expects to provide credible and scientific evidence for the efficacy and safety of HPSR on outcomes for patients with AF.

**Registration number:**

10.17605/OSF.IO/WAEBN.

## 1 Introduction

Radiofrequency ablation is a widely used rhythm control strategy in the management of atrial fibrillation (AF). Since the pioneering work of Haissaguerre et al, pulmonary vein isolation has become the standard strategy for catheter ablation of atrial fibrillation.^[1,2]^ The aim of pulmonary vein isolation is to electrically separate the pulmonary veins from the left atrium at the level of the pulmonary vein ostia. Although alternative energy sources such as low temperature, laser power and microwaves are available, radiofrequency ablation remains the most widely used source of energy for point-to-point ablation to wide antral pulmonary vein isolation.^[3,4]^

Experimental studies have shown that applying high-power short-duration radiofrequency (HPSR) increases the resistance but reduces the conductive heat, resulting in a lesion depth similar to that of conventional energy delivery (25–30 W).^[5,6]^ Recent studies have shown that HPSR can form wider, shallower lesions compared to conventional methods, although the lesions are similar in size.^[7–9]^ Theoretically, HPSR may reduce the length of surgery without increasing the incidence of complications compared to conventional radiofrequency. Actually, it has been reported that HPSR may increase surgery-related complications, such as steam popping, pericardial effusion, and gastrointestinal discomfort.^[10]^

HPSR has been supposed to increase efficacy and minimize deep tissue injury. However, studies comparing data between the HPSR and low power longer duration therapy were limited and inconsistent.^[6–8]^ Therefore, we conduct a high-quality systematic review and meta-analysis to assess the efficacy and safety of HPSR on outcomes for patients with AF. It was hypothesized that the HPSR may enhance the durability of the isolated pulmonary vein and procedural outcome of in patients with AF.

## 2 MAterials and methods

### 2.1 Data sources and search strategy

This systematic review and meta-analysis will be conducted according to the Preferred Reporting Items for Systematic Review and Meta-Analysis (PRISMA) guidelines.^[11]^ The online literature is searched using the following combination of medical subject heading terms: “high-power” OR “high power” AND “radiofrequency” AND “atrial fibrillation.” MEDLINE (PubMed), EMBASE (OVID), Cochrane Central Register of Controlled Trials (CENTRAL), and Web of Science (ISI database) will be searched without any language restrictions. The reference lists of the included studies will be also checked for additional studies that are not identified with the database search. The systematic review protocol has been registered on Open Science Framework registries. Ethical approval and patient consent are not required because this study is a literature-based study.

### 2.2 Eligibility criteria

Study included in our meta-analysis have to meet all of the following inclusion criteria:

1. all clinical trials to assess the efficacy and safety of HPSR in the treatment of atrial fibrillation will be considered eligible for analysis;
2. comparing HPSR with low power longer duration therapy;
3. reporting the available data on the following outcomes: procedure time, ablation time, fluoroscopy time, atrial arrhythmias recrudescence, first-pass pulmonary vein isolation, and major complications.

Studies with overlapping data or insufficient data to calculate or extract effect estimates will be excluded. Case reports, biochemical trials, letters, and reviews will also be eliminated.

### 2.3 Data extraction

A standard data extraction form will be used independently by 2 reviewers to retrieve the relevant data from the articles. These variables included author, study design, sample size, publishing date, population, type of interventions, follow-up, and outcomes. The outcome measures include procedure time, ablation time, fluoroscopy time, atrial arrhythmias recrudescence, first-pass pulmonary vein isolation, and major complications. Data extraction will be performed independently, and any conflict will be resolved before final analysis. If data are not presented in the original article, corresponding authors will be contacted to acquire the missing data. Otherwise, the results will be extracted manually from the published figures. If necessary, we will abandon the extraction of incomplete data.

### 2.4 Statistical analysis

The present study will be performed by Review Manager Software (RevMan Version 5.3, The Cochrane Collaboration, Copenhagen, Denmark). Mean differences with a 95% confidence interval are assessed for continuous outcomes. *P* < .05 is set as the significance level. We will examine heterogeneity graphically using forest plots and statistically by calculating the *I*^2^ statistic, which describes the percentage of the variability in effect estimates that is due to heterogeneity rather than sampling error (chance). We consider an *I*^2^ statistic greater than 50% to be substantially heterogeneous. All outcomes will be pooled on random-effect model. The Z test will be used to assess the overall effect. A meta-analysis will be conducted when 4 or more trials reported an outcome of interest. Subgroup analyses will be planned by different follow-up periods. We will also conduct the sensitivity analysis to evaluate whether any single study has the weight to skew on the overall estimate and data. Begg funnel plot will be used to assess publication bias. If publication bias exists, the Begg funnel plot is asymmetric.

### 2.5 Quality assessment

In order to achieve a consistency (at least 80%) of risk of bias assessment, the risk of bias assessors will preassess a sample of eligible studies. Results of the pilot risk of bias will be discussed among review authors and assessors. Two independent reviewers will assess the risk of bias of the included studies at study level. We will follow the guidance in the latest version of Cochrane Handbook for systematic reviews of interventions when choosing and using tools to assessing risk of bias for randomized trials (version 2 of the Cochrane risk of bias tool for randomized trials, RoB 2) and nonrandomized trials (the Risk Of Bias In Nonrandomized Studies of Interventions, ROBINS-I tool). Any disagreements will be discussed and resolved in discussion with a third reviewer. Studies with high risk of bias or unclear bias will be given less weight in our data synthesis.

## 3 Discussion

Radiofrequency ablation is a widely used rhythm control strategy in the management of AF. However, studies comparing data between the HPSR and low power longer duration therapy were limited and inconsistent.^[6–8]^ Therefore, we conduct a high-quality systematic review and meta-analysis to assess the efficacy and safety of HPSR on outcomes for patients with AF. It was hypothesized that the HPSR may enhance the durability of the isolated pulmonary vein and procedural outcome of in patients with AF. This study expects to provide credible and scientific evidence for the efficacy and safety of HPSR on outcomes for patients with AF.

## Author contributions

**Conceptualization:** Fei Wang.

**Data curation:** Peng Zhang, Ling Ma.

**Formal analysis:** Peng Zhang, Ling Ma.

**Funding acquisition:** Liang Shi.

**Investigation:** Peng Zhang, Ling Ma.

**Methodology:** Ling Ma, Fei Wang.

**Project administration:** Liang Shi.

**Resources:** Fei Wang.

**Software:** Peng Zhang, Ling Ma.

**Supervision:** Liang Shi.

**Validation:** Fei Wang.

**Visualization:** Peng Zhang, Fei Wang.

**Writing – original draft:** Peng Zhang.

**Writing – review & editing:** Liang Shi.
